# Antigen Retrieval and Its Effect on the MALDI-MSI of Lipids in Formalin-Fixed Paraffin-Embedded Tissue

**DOI:** 10.1021/jasms.0c00208

**Published:** 2020-07-17

**Authors:** Vanna Denti, Isabella Piga, Sonia Guarnerio, Francesca Clerici, Mariia Ivanova, Clizia Chinello, Giuseppe Paglia, Fulvio Magni, Andrew Smith

**Affiliations:** † Clinical Proteomics and Metabolomics Unit, Department of Medicine and Surgery, University of Milano-Bicocca, Vedano al Lambro 20854, Italy; ‡ Biomolecular Sciences Research Centre, Sheffield-Hallam University, City Campus, Howard Street, Sheffield S1 1WB, United Kingdom

**Keywords:** Lipids, Lipidomics, FFPE tissue, MALDI-MS, Imaging mass spectrometry

## Abstract

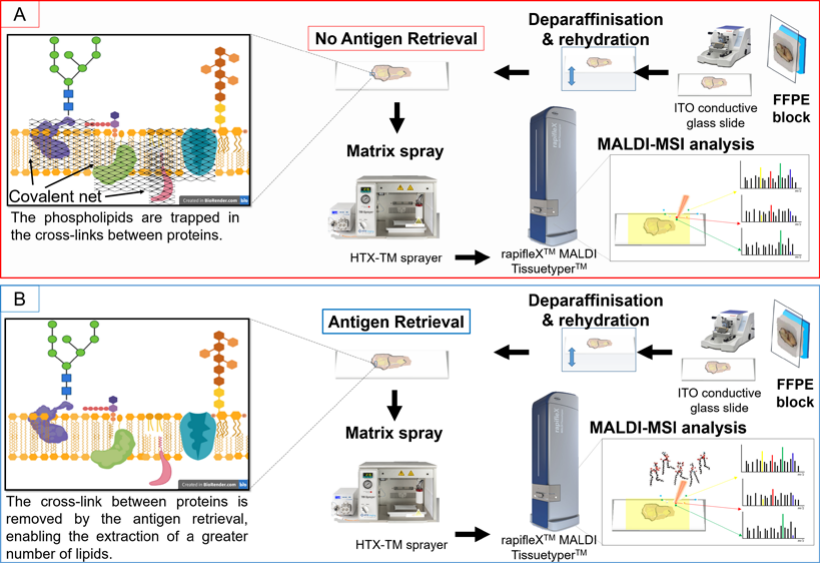

Formalin-fixed paraffin-embedded (FFPE) tissue represents the primary source of clinical tissue and is routinely used in MALDI-MSI studies. However, it is not particularly suitable for lipidomics imaging given that many species are depleted during tissue processing. Irrespective, a number of solvent-resistant lipids remain, but their extraction may be hindered by the cross-link between proteins. Therefore, an antigen retrieval step could enable the extraction of a greater number of lipids and may provide information that is complementary to that which can be obtained from other biomolecules, such as proteins. In this short communication, we aim to address the effect of performing antigen retrieval prior to MALDI-MSI of lipids in FFPE tissue. As a result, an increased number of lipid signals could be detected and may have derived from lipid species that are known to be implicated in the lipid–protein cross-linking that is formed as a result of formalin fixation. Human renal cancer tissue was used as a proof of concept to determine whether using these detected lipid signals were also able to highlight the histopathological regions that were present. These preliminary findings may highlight the potential to enhance the clinical relevance of the lipidomic information obtained from FFPE tissue.

## 1 Introduction

Formalin fixation and paraffin embedding (FFPE) of tissue represents the gold standard for specimen preservation in pathology units, enabling the long-term storage of samples and the generation of large tissue banks.^1,2^ This form of preservation initiates the process of amine–thiol cross-linking, and the resulting methylene bridges inactivate enzymatic activity, stabilizing the biomolecules within the tissue.^3^ As a result, FFPE tissue specimens are employed for routine diagnostic assessment and also represent a valuable source of molecular information that can be exploited for biomarker discovery.^1,2,4^

Although matrix-assisted laser desorption/ionization mass spectrometry imaging (MALDI-MSI) is now routinely used to explore the spatial distribution of proteins in FFPE tissue, the detection of lipid species is somewhat more challenging.^5,6^ This is primarily related to the phenomenon of many lipid species being depleted from the tissue by the use of paraffin wax and organic solvents during the processing of the tissue.^7^ Nevertheless, recent studies employing MALDI-MSI^8^ and Fourier transform infrared spectroscopy^9^ have provided evidence to suggest that some of these solvent-resistant lipid species are maintained in FFPE tissues and may provide diagnostically relevant information.

Among these solvent-resistant lipids, a proportion of them may be confined within the covalent network of proteins formed during fixation and may not be efficiently extracted. Therefore, an antigen retrieval (AR)^10^ step prior to MALDI-MSI analysis, as is traditionally performed in protocols for the MSI of tryptic peptides,^11−14^ may enable access to these lipids and increase the coverage of species detected.

In this work, we explore the effect of AR on the MALDI-MSI detection of lipid species in FFPE tissue, observing an increased number of lipid species in human renal tissue that also better reflected the heterogeneous morphology of the specimens. These preliminary results indicate that AR may help to enhance the degree of lipidomic information that can be obtained from FFPE tissue using MALDI-MSI.

## 2 Experimental Section

### 2.1 Specimen Selection

The FFPE blocks selected for this study included normal kidney and clear cell renal cell carcinoma (ccRCC), both from nephrectomy specimens performed for neoplasia at the University of Milano-Bicocca, San Gerardo Hospital, Monza, Italy.^15^ The appropriate Ethical Committee approved the collection of these specimens, and informed consent was obtained from all participants.

### 2.2 Fixation and Cutting

Fixation time was set at 24 h following the surgical procedure, as previously described. Four-micron-thick sections were cut and mounted onto conductive glasses coated with indium tin oxide (Bruker Daltonik GmbH, Bremen, Germany).^1^ The slides were stocked at room temperature until the day of the analysis.

### 2.3 Sample Preparation

For positive ion imaging, consecutive tissue sections from five FFPE blocks were prepared using a protocol without an antigen retrieval step (A) and with an antigen retrieval step (B). The latter protocol (B) was then used to analyze another tissue that was characterized by more complex histopathological features. Finally, an additional two cases of ccRCC were analyzed using protocols A and B for preliminary negative ion imaging analysis.

In both protocols (A and B), the slides were first deparaffinized and rehydrated by performing consecutive washes with toluene (2 × 5 min), isopropyl alcohol (1 × 5 min), 100% ethanol (1 × 5 min), 90% ethanol (1 × 5 min), and 70% ethanol (1 × 3 min).

In protocol A, the tissue was brought to hydration by washing in HPLC grade H_2_O for 2 min prior to matrix application. In protocol B, the slides were washed twice in HPLC grade H_2_O for 3 min each. The antigen retrieval was performed in a bath of 10 mM citric acid buffer at 97 °C for 45 min before being brought to hydration by washing in HPLC grade H_2_O for 2 min prior to matrix application.

### 2.4 Matrix Application

For positive ion imaging, equimolar quantities of aniline and 10 mg/mL α-cyano-4-hydroxycinnamic acid (ANI-CHCA)^16^ were dissolved in a 70% methanol solution with 0.1% trifluoroacetic acid. This matrix solution was deposited using the HTX TM-Sprayer (HTX Technologies, LLC), with the following parameters: temperature = 75 °C; number of passes = 2; flow rate = 0.06 mL/min; velocity = 1200 mm/min; track spacing = 1.2 mm; pressure = 5 psi.

For negative ion imaging, 10 mg/mL 9-aminoacrdine was dissolved in a 70% methanol solution and deposited using the HTX TM-Sprayer (HTX Technologies, LLC), with the following parameters: temperature = 85 °C; number of passes = 6; flow rate = 0.2 mL/min; velocity = 1100 mm/min; track spacing = 2 mm; pressure = 10 psi.

### 2.5 MALDI-MSI

All analyses were performed using a rapifleX MALDI Tissuetyper mass spectrometer (Bruker Daltonics, Bremen, Germany) equipped with a Smartbeam 3D laser. External calibration was performed using red phosphorus clusters in the *m*/*z* range of 0 to 2000.^17^ Mass measurements in positive ion reflectron mode were acquired in the *m*/*z* range of 420 to 820, whereas mass measurements in negative ion reflectron mode were acquired in the *m*/*z* range of 500 to 900. In both instances, 400 shots were accumulated for each spectrum and the matrix suppression deflection was set to *m*/*z* 400. The samples were rastered at a lateral resolution 50 × 50 (*x*,*y*) μm with a laser scan range of 44 μm per pixel.

### 2.6 On-Tissue MALDI-MS/MS

A single precursor ion was selected by using the smallest precursor ion selector window possible and dissociated using LID-LIFT technology,^18^ with the laser energy being set within a range of 40–70%. This process was performed until an MS/MS spectrum was obtained from the accumulation of ∼100,000 laser shots.

### 2.7 Histological Staining

Following MALDI-MS analysis, tissue sections were washed with ethanol (70 and 100%) and the slides were stained using hematoxylin and eosin (H&E).^19^ The slides were converted to digital format by scanning using a ScanScope CS digital scanner (Aperio, Park Center Dr., Vista, CA, USA).^1^

### 2.8 Data Processing

Data files containing the individual spectra of each entire measurement region were imported into SCiLS Lab MVS 2019b software (http://scils.de/; Bremen, Germany) for spectra preprocessing and to annotate regions of interest (ROIs). Average spectra of those samples analyzed without AR (protocol A) or with AR (protocol B) were generated.^1,19^ These average spectra were then imported into mMass (version 5.5.0, http://www.mmass.org), where peak picking (S/*N* ≥ 5) was performed.^20,21^Finally, a reference list of α-CHCA cluster signals^22^ was imported into mMass, and if a matrix peak was matched with a mass tolerance of ≤50 ppm, it was removed from the final peak list.

For lipid identification, product ions in the acquired MALDI-MS/MS spectra were annotated within mMass after being cross-referenced with known product ions generated by the fragmentation of different phospholipid species.^23−25^ Then, identifications were assigned by matching the mass of the precursor ion with lipids listed in the METLIN,^26^ HMDB,^27^ and Lipid Maps^28^ databases.

## 3 Results and Discussion

**Figure 1:**
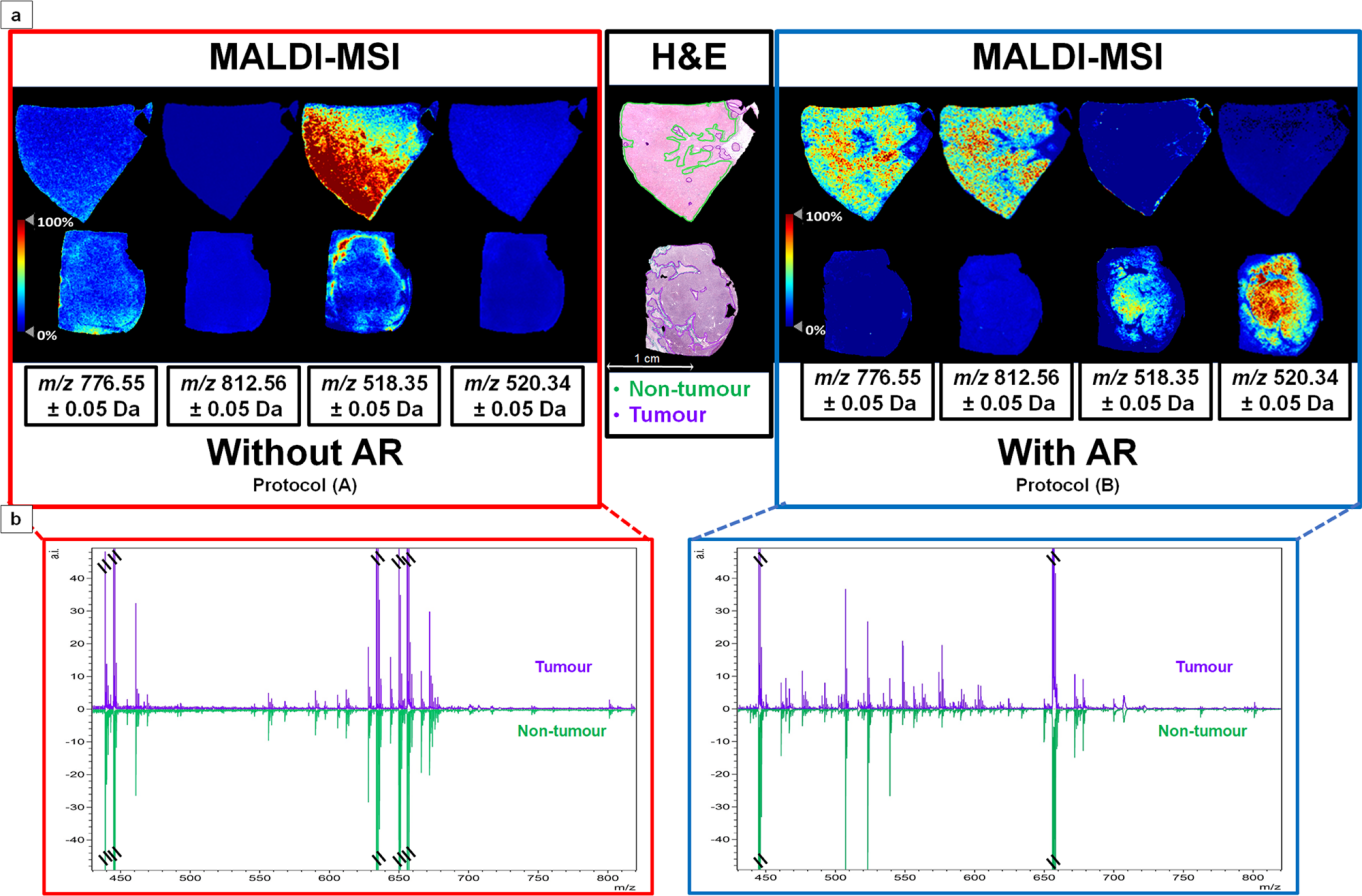
(a) Example MALDI-MS images of consecutive sections analyzed without antigen retrieval (protocol A; left) and with antigen retrieval (protocol B; right), highlighting the localization of two signals. In samples prepared with protocol B, the signals at *m*/*z* 518.35 (LPC18:3) and at *m*/*z* 520.34 (LPC 18:2) had a higher intensity in the tumor region, as indicated by a purple line in the H&E image (center), whereas the signal at *m*/*z* 776.55 (PC(34:4) + Na) and at *m*/*z* 812.56 (PS 36:1) had a higher intensity in the nontumor region, as indicated by the green line in the H&E image. The same signals were not detected in the samples prepared with protocol A. (b) MALDI-MS profiles of the two regions highlighted in the H&E image. On the left (red rectangle) are average spectra from the tumor (purple) and the nontumor (green) regions analyzed using protocol A. On the right (blue rectangle) are average spectra from the tumor (purple) and the nontumor (green) regions analyzed using protocol B. Those peaks assigned to α-CHCA matrix clusters are indicated by the \\ symbols.

When assessing the effect of antigen retrieval on the detection of positive ion lipid species in FFPE tissue, the total number of peaks detected in the tissue treated without AR (protocol A) was 74, whereas with AR (protocol B), a total number of 129 peaks were detected. Moreover, we observed that the signals detected with protocol B enabled the tissue to be separated into the tumoral and nontumoral regions indicated by the H&E staining (Figure 1a), with these signals not being detected when using protocol A. This was also evident in the average spectra generated for the tumor and nontumor regions (Figure 1b), with distinct differences between them only being observed when antigen retrieval was performed. On the contrary, we observed that the signals obtained with protocol A did not reflect the morphology of the samples analyzed. This effect is further highlighted in Supplemental Figure S1. Given the low abundance of the lipids extracted by the matrix, the differences in signal intensities could be more related to tissue density and variations on extraction efficiency rather than any histopathological differences within the tissue.

**Figure 2:**
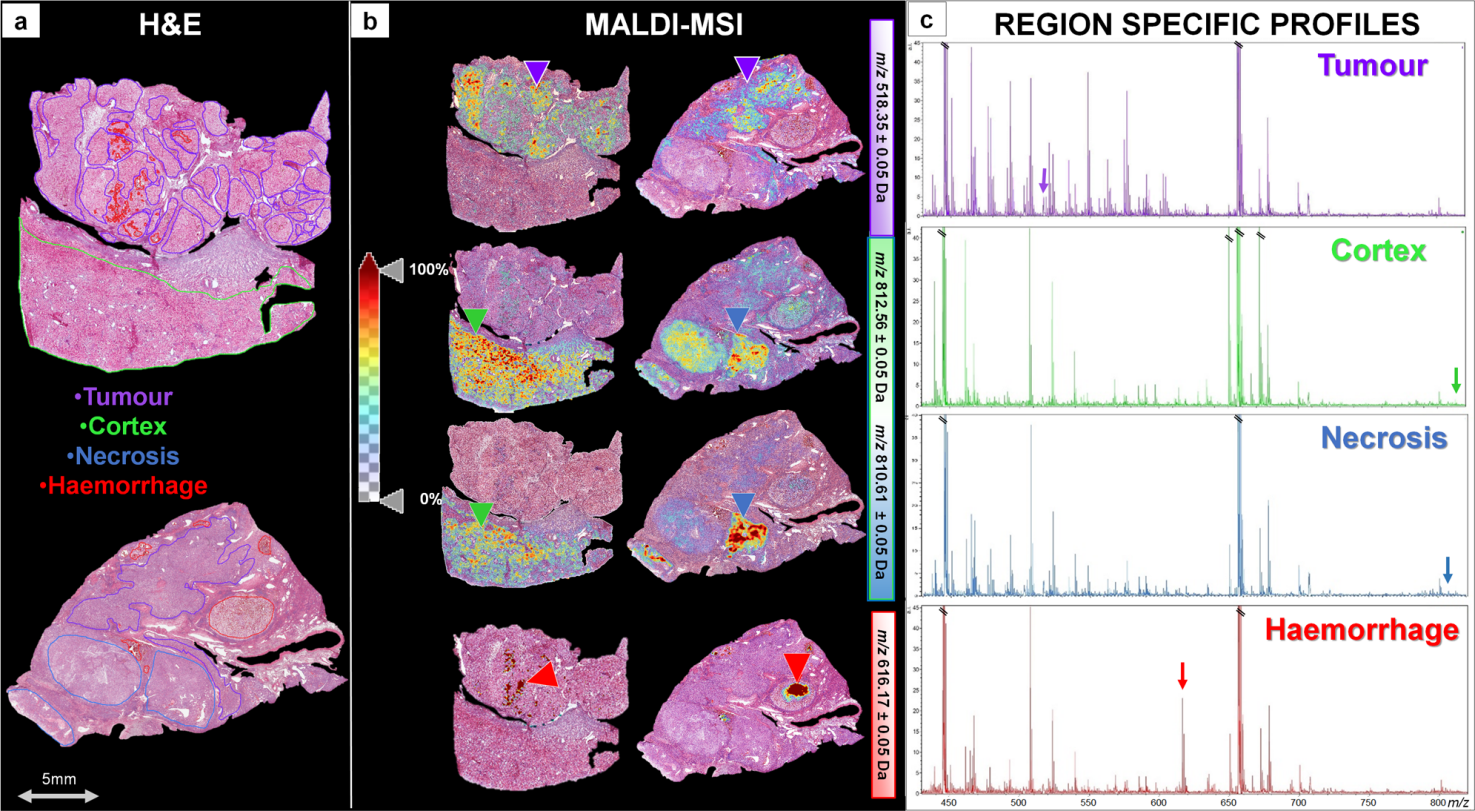
(a) H&E-stained images of the two additional tissue sections analyzed. The regions of interest (ROIs) are annotated with different colors. (b) MALDI-MS images of four *m*/*z* signals localized in specific regions of three different samples and indicated by triangles. Color scale bars are provided to represent relative signal intensity. The *m*/*z* 518.35 (LPC 18:3) is more intense in the tumor regions, whereas *m*/*z* 812.56 (PS 36:1) and 810.61 (PC(36:4)+Na) are of higher intensity in both the cortex and necrotic regions. Finally, *m*/*z* 616.17 (heme) is present solely in the hemorrhagic regions. (c) Average spectra of the ROIs indicated in the H&E-stained image. Arrows indicate the peaks highlighted in MALDI-MS images.

In order to further evaluate the possibility to detect regiospecific profiles when performing an AR step, two additional specimens, which were characterized by more complex histopathology, were analyzed. The tumoral, cortical, necrotic, and hemorrhagic regions present within these tissue sections (Figure 2a) could also be spatially resolved using individual *m*/*z* signals at 518.35, 812.56, 810.61, 788.64, and 616.17 (the latter corresponding with a fragment of the heme group^29,30^) (Figure 2b). These regiospecific signatures were also evident in the average profiles generated from these ROIs (Figure 2c) and support the proposal that it can be possible to still detect clinically relevant lipid species in FFPE samples, despite the loss of a large proportion during tissue processing.

In order to gain an initial understanding of the phospholipid classes that AR renders more accessible, on-tissue MALDI-MS/MS was performed on the primary signals whose distribution was in agreement with the histology of the tissue. An overview of the product ions generated and the resulting lipid identifications are provided in Supplemental Table S1a. Briefly, a number of different lipid species were identified, including phosphatidylcholine (PC), as well as their lyso variants (LPC), and sphingomyelin (SM), with these lipid species already reported to be readily detected in fixed tissue.^31,32^ Although it seems that PC and SM are not directly involved in the amine–thiol cross-linking, those that remain after the process of paraffin embedding are part of the membrane protein–lipid complex. As such, we could speculate that their extraction is rendered more efficient without the steric hindrance caused by the presence of large protein complexes.^33^ Moreover, a phosphatidylserine (PS) lipid was also identified, and this can be particularly relevant given that the headgroups of PS lipids react directly with formalin to promote the formation of cross-links between themselves and other amine-containing molecules.^7^ Thus, this finding may serve as further evidence of the effect that AR has on the detection of positive ion lipid species in FFPE tissue.

Preliminary analysis in negative ion mode was also performed in order to evaluate the effect of AR on the detection of further phospholipid classes whose ionization in positive ion mode can be hindered by the presence of PC.^34^ In this instance, lipid signals whose spatial distribution was coherent with tissue morphology were detected using both protocols (A and B) (Supplemental Figure S2). Among these, some phosphatidic acid, phosphatidylglycerol, and phosphatidylinositol species were detected both without and with AR (Supplemental Table S1b) and is in accordance with previous observations where negative ion MALDI-MS imaging of FFPE tissue was performed.^8^ Whereas there were further *m*/*z* signals detected exclusively in the tissue prepared with AR, their identification was hindered by difficulties encountered in precursor ion selection and the resulting complexity of the on-tissue MS/MS spectra (data not shown). Accordingly, a more systematic approach, employing high mass accuracy MS and ion mobility,^35^ should be considered in order to obtain a more comprehensive overview of the solvent-resistant lipid classes that become accessible as a result of performing antigen retrieval.

## 4 Conclusions

Whereas many nonpolar lipid species are depleted from FFPE specimens during the processing of the tissue, some solvent-resistant lipids may remain present and locked in the cross-linked network that arises as a result of formalin fixation.^36^ The introduction of an antigen retrieval step seems to increase the number of these solvent-resistant lipids (PC, LPS, SM, and PS) that can be detected in FFPE tissue samples when performing MALDI-MSI. We have observed that the protocol is able to maintain the specific localization of these lipids signals in different areas of ccRCC tissue, used as testing a specimen, according to their histopathological features, and by exploiting this lipidomic information, it may then be possible to obtain clinically relevant information. In conclusion, these highly promising findings indicate that it may be possible to detect alterations in the spatial lipidome of FFPE tissue, opening up new possibilities for spatial omics investigations of disease.

## Supporting Information Available

Supplemental Table S1 (PDF)
Supplemental Figure S1 (PDF)
Supplemental Figure S2 (PDF)
