# Supplementary material for: Antigen Retrieval and Its Effect on the MALDI-MSI of Lipids in Formalin-Fixed Paraffin-Embedded Tissue
Source: J Am Soc Mass Spectrom. 2020 Jul 17;31(8):1619–24. doi: 10.1021/jasms.0c00208 (PMC8009503; doi:10.1021/jasms.0c00208)
Supplement: Supplementary file 1 — js0c00208_si_001.pdf [file js0c00208_si_001.pdf]

Antigen retrieval and its effect on the MALDI-MSI of lipids in formalin-fixed paraffin-embedded tissue

Vanna Denti[a], Isabella Piga[a], Sonia Guarnerio[b], Francesca Clerici[a], Mariia Ivanova[a], Clizia Chinello[a], Giuseppe Paglia[a], Fulvio Magni[a], Andrew Smith[a]

[a] Clinical Proteomics and Metabolomics Unit, Department of Medicine and Surgery, University of Milano-Bicocca, Vedano al Lambro, Italy
[b] Biomolecular Sciences Research Centre, Sheffield-Hallam University, City Campus, Howard Street, Sheffield, UK

Supplemental table

a)

PRECURSOR *m/z*	*m/z* OF PRODUCTS (POSTIVE)	ID	PROTOCOL
518.35	104 184 335 459 499	LPC(18:3)	B
520.34	86 104 184 258 502	LPC(18:2)	B
522.46	86 104 184 504	LPC(18:1)	B
552.69	86 104 184 258 534	LPC(20:0)	B
585.37	147 402 526	SM(34:1)+Na	B
776.55	147 184 593 717	PC(34:4)+Na	B
782.56	86 184 599 724	PC(36:4)	B
782.56	86 147 184 599 724	PC(34:1)+Na	B
799.71	184 617 723	SM(41:2)	B
810.61	86 104 147 184 470 628 752	PC(36:4)+Na	B
812.56	208 606 628 714 726	PS(36:1)	B
812.63	86 147 184	PC(36:0)+Na	B

b)

PRECURSOR *m/z*	*m/z* OF PRODUCTS (NEGATIVE)	ID	PROTOCOL
553.38	79 97 153 171 227 241 255 297 303 315 391 483	PG(20:0)	A,B
571.29	79 97 153 179 241 255 315 391	LPI(16:0)	A
591.48	79 97 143 153 279 297 311 447 465	PA(20:0/8:0)	A,B
599.39	79 97 153 241 283 315 419	LPI(18:0)	A
619.29	79 97 153 241 303 315 439	LPI(20:4)	A
673.49	79 255 281 391 409 417 435	PA(16:0/18:1)	B
766.54	79 97 153 255 303 480	PC(36:4)[-CH3]	B
863.57	79 97 153 241 281 283 419 581 599	PI(18:0/18:0)	A,B
885.53	79 97 153 223 241 259 283 303 419 439 581 599 619	PI(20:4/18:0)	A

Supplemental Table.
a) Overview of the positive ion phospholipid species identified by LID-LIFT™ fragmentation of tissue ions detected by MALDI-MS imaging. The right-hand column indicates with which protocol the lipid ion is observed
b) Overview of the negative ion phospholipid species identified by LID-LIFT™ fragmentation of tissue ions detected by MALDI-MS imaging. The right-hand column indicates with which protocol the lipid ion is observed
