# Supplementary material for: Antigen Retrieval and Its Effect on the MALDI-MSI of Lipids in Formalin-Fixed Paraffin-Embedded Tissue
Source: J Am Soc Mass Spectrom. 2020 Jul 17;31(8):1619–24. doi: 10.1021/jasms.0c00208 (PMC8009503; doi:10.1021/jasms.0c00208)
Supplement: Supplementary file 3 — js0c00208_si_003.pdf [file js0c00208_si_003.pdf]

Antigen retrieval and its effect on the MALDI-MSI of lipids in formalin-fixed paraffin-embedded tissue

Vanna Denti[a], Isabella Piga[a], Sonia Guarnerio[b], Francesca Clerici[a], Mariia Ivanova[a], Clizia Chinello[a], Giuseppe Paglia[a], Fulvio Magni[a], Andrew Smith[a]

[a] Clinical Proteomics and Metabolomics Unit, Department of Medicine and Surgery, University of Milano-Bicocca, Vedano al Lambro, Italy
[b] Biomolecular Sciences Research Centre, Sheffield-Hallam University, City Campus, Howard Street, Sheffield, UK

Supplemental figure 2

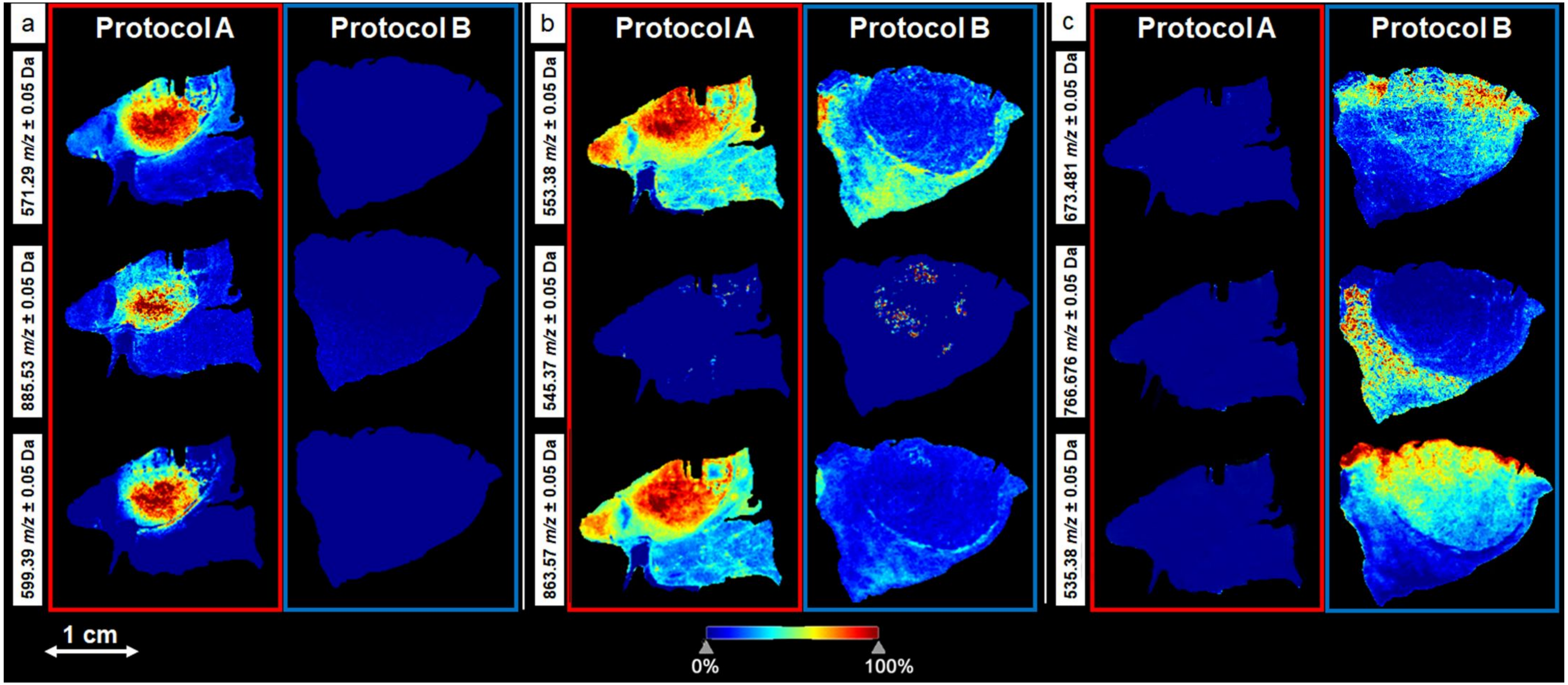

Supplemental Figure 2.
MALDI-MS images of negative ions detected in ccRCC samples with Protocol A or Protocol B
a) Example ion images demonstrating the tissue distribution of *m/z* signals detected exclusively in samples prepared without AR (protocol A)
b) Example ion images demonstrating the tissue distribution of *m/z* signals detected in samples prepared without AR and with AR (both protocols A and B)
c) Example ion images demonstrating the tissue distribution of *m/z* signals detected exclusively in samples prepared with AR (protocol B)
Both scale and intensity bars are provided.
